# Low dielectric resins derived from hyperbranched carbosilane oligmers functionalized by benzocyclobutene groups

**DOI:** 10.1080/15685551.2021.2003556

**Published:** 2021-12-09

**Authors:** Xian Li, Yawen Huang, Xu Ye, Quan Long, Wen Yuan, Li Fan, Qiuxia Peng, Jiajun Ma, Junxiao Yang

**Affiliations:** a School of Materials Science and Engineering, Southwest University of Science and Technology, Mianyang China; b State Key Laboratory of Environmental-friendly Energy Materials, Southwest University of Science and Technology, Mianyang China; c School of Adult and Network Education, Southwest University of Science and Technology, Mianyang China; d Sichuan College of Traditional Chinese Medicine, Mianyang China; e School of National Defense Science and Technology, Southwest University of Science and Technology, MianyangChina

**Keywords:** Polycarbosilanes, hyperbranched carbosilane oligomers, benzocyclobutene, resin

## Abstract

Polycarbosilanes have been considered as potential materials used in electronic packaging and circuit boards owing to their excellent low-dielectric performance. In this work, we prepared new hyperbranched carbosilane oligomers (HCBOs) which were functionalized by benzocyclobutene (BCB) groups. HCBOs can be thermally cured to produce transparent (HCBRs) with low dielectric constant and high thermostability.

## 1 Introduction

Low-dielectric polymeric materials have demonstrated promising application in circuit boards, microwave transmitting, etc. [1]. The structures of polymeric materials determine the ultimate low-dielectric performance in high degree. In terms of this point, in past decades, researchers have prepared a large number of low-dielectric polymers, including epoxy resins, polyimides, polyphenyl oxide, polybenzoxazine, silicon resins, etc. [2–5]. The strategy of structure design for low-dielectric polymers is well established. The high-performance low-dielectric materials are limited to a few kind of polymers, including carbohydrogen resins (typically aryl resins and BCB resins [6,7]), polycarbosilane resins [8,9], etc. Polycarbosilanes are a family of polymers with the backbone constructed by Si-C bonds. Owing to the low polarity of Si-C bonds, polycarbosilanes were found to exhibit low k values and high thermostability while preserving high mechanical performance. Thus, polycarbosilanes have shown great potential in low-dielectric application. Our groups have done a series of research on low dielectric BCB resins in past few years [10–14]. Recently, we prepared several low dielectric resins which combine the structure of BCB and polycarbosilanes. The *k* of these resins can reach to around 2.7.

Hyperbranched polymers are synthesized by the polymerization of the monomers with two A functional groups and one B functional group. As a result, the hyperbranched polymers possess a large number of side chains, thus increasing free molecular volume between inter-chains [15–17]. The use of hyperbranched structures in low-dielectric polymers has been reported. However, they are generally used as fillers [18], because the hyperbranched polymers exhibit poor film-forming property. In our previous work, the BCB groups were functionalized on hyperbranched polymers to realize the cross-linking and to improve the performance of polymers [19]. The hyperbranched polymers (BCBPCS) were prepared from the (Chrolomethyl)trimethoxysilane by Grignard reaction. As a result, a number of Si-O bonds were inevitably formed in the hyperbranched polymers.

In this work, we employed chloromethyltrichlorosilane as monomer to prepare hyperbranched carbosilane oligomers (HCBOs). In this way, more Si-C bonds rather than Si-O bonds could be formed in HCBOs. Subsequently, BCB was functionalized on HCBOs [19,20]. Our experiments indicated that HCBOs were curable and HCBRs can be shaped. HCBRs have relatively high C/Si ratio, low dielectric constant of 2.72 and T_5%_ of 458.1°C. The structures, curing behavior and performance of HCBRs were investigated in detail.

## 2 Experimental section

### 2.1 Materials

Chloromethyltrichlorosilane (97.0%) was purchased from Shanghai Tangui Advanced Material Technology Co., Ltd. 4-Bromobenzocyclobutene (97.0%) was synthesized in the laboratory according to reported methods [21,22]. Tetrahydrofuran (THF, 98%) was purchased from J&K Scientific (China). Toluene (98%), ethanol, ethyl acetate and sodium sulfate were purchased from Chengdu Kelong Chemical Reagent Co., Ltd. Tetrahydrofuran was dried and freshly distilled under nitrogen (N_2_) atmosphere in prior to use. Other solvents were used without further purification.

### 2.2 Characterization

Fourier transform infrared (FTIR) spectroscopy measurements at 4000–400 cm^−1^ were conducted on a Nicolet FTIR IS5 spectrophotometer at room temperature. The samples were prepared for analysis by casting solution on KBrplates.^1^Hand^13^C NMR spectra were recorded on a Bruker Avance-600 using deuterated chloroform (CDCl_3_) as the solvent and tetramethylsilane as the internal reference. Differential scanning calorimeter (DSC) was conducted under nitrogen at a rate of 10 °C/min on a TA instrument 2100. Thermal gravimetric analysis (TGA) was performed at room temperature to 800 °C on a TA 2100 thermogravimetric analyzer in a nitrogen atmosphere at a heating rate of 10 °C/min. Nanoindentation was performed with an Agilent Nano Indenter G200 (U9820A, Agilent Technologies., Inc.) at room temperature. Five measurements were conducted per sample as follows: a strain rate of 0.2 s^−1^ was constantly maintained during the addition of the load until the indenter reached a depth of 2000 nm on the surface of the scaffold. The load maintained at a maximum value for 10 s. The surface morphology and roughness of the sample film were observed by using the PicoStation white light interferometry system (PicoStation 2000, Single field of view range: 1*1 mm, measurement resolution: 0.1 nm, measurement accuracy: 1 nm). The dielectric constant (*ε*_r_) and dielectric loss of the resin were measured on an Agilent 4294 A Impedance Analyzer with varying frequencies at ambient temperatures. (After the samples were thoroughly dried under vacuum, the surface of the both sides was coated with silver paint to form electrode. The final data were obtained from the average of the results in four of the same samples.) The dielectric constant was calculated by the following equation:

$$\varepsilon_{r}=\left(C\times d\right)/\left(\varepsilon_{0}\times S\right)$$

Where *C, d* and *S* denote respectively the capacitance, thickness and electrode area of the materials. *ε*_0_ denotes the permittivity of free space and equals 8.854 × 10^−12^ F/m.

### 2.3 Synthesis of HCBOs

In a 100 mL round bottom flask, magnesium (1.06 g, 44 mmol) and a grain of Iodine were added. Under the atmosphere of nitrogen, the mixture of chloromethyltrichlorosilane (1.84 g, 10 mmol) and THF (3.83 g, 60 mmol) was added dropwise into the flask at a rate of 1 drop/10 s. Then, the reaction mixture was heated to 68°C and stirred continuously for 7–8 h. A solution of 4-BrBCB (5.49 g, 30 mmol) in THF (10.98 g, 153 mmol) was slowly added into the front reaction mixture with same rate at ambient temperature. Then, the reaction mixture was heated to 68°C and stirred continuously for 8 h. After the reaction, the mixture was cooled to room temperature and quench the reaction. Ethanol was added to the mixture to precipitate and separate. The product was extracted with toluene and dried in anhydrous sodium sulfate. After removing the solvents by rotary evaporation, the organic layer was purified by silica-gel column chromatography (toluene/n-hexane) to obtain the pale yellow jelly. ^1^H NMR (600 MHz, CDCl_3_) δ: 6.98–7.43 (m, ArH), 3.10–3.25 (m, – CH_2_CH_2_-), 1.00–1.89 (m, – Si-CH_2_-Si-). ^13^C NMR (151 MHz, CDCl_3_) δ: 147.34, 145.54, 132.76, 128.07, 126.32, 121.94, 29.88, 14.04.

### 2.4 Preparation of the cured polymer resins

The sample was heated at 180 °C for 4 h and then cured stepwise at 200 °C for 4 h, 220 °C for 2 h and 250 °C for 2 h in a glass mold under nitrogen atmosphere. Then reduce to room temperature at 50 °C/h. The yellow curing product can be obtained which was insoluble and infusible.

## 3 Results and discussion

### 3.1 Preparation of HCBOs and derived cured resins

**Scheme 1.**
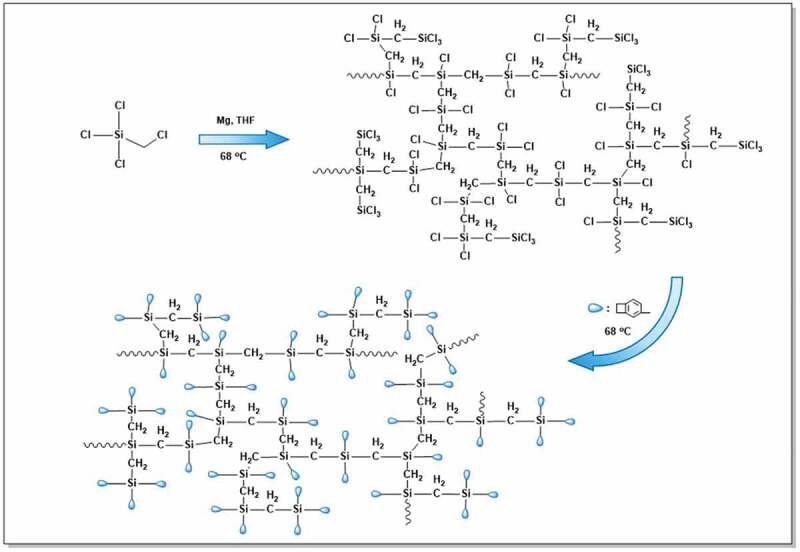
Synthetic routes of HCBOs

The synthetic route to HCBOs is depicted in Scheme 1. First, the – CH_2_Cl group in CH_2_ClSiCl_3_ reacts with Mg through Grignard reaction to generate intermediate, and then the intermediate reacts with Si-Cl in a coupling reaction. As each CH_2_ClSiCl_3_ molecule has three groups of Si-Cl, branched polycarbosilane will be formed with the increase of reaction degree. Subsequently, the unreacted group of Si-Cl reacted with the Grignard reagent (BCBMgBr) to introduce BCB into the polymer. The HCBOs was formed in a complex 3D network structure. The obtained HCBOs oligomers were soluble in most organic solvents such as toluene, THF, and dichloromethane. In addition, HCBOs oligomers could be easily spin-coated to form a film, suggesting that this product have good processability.

**Figure 1.**
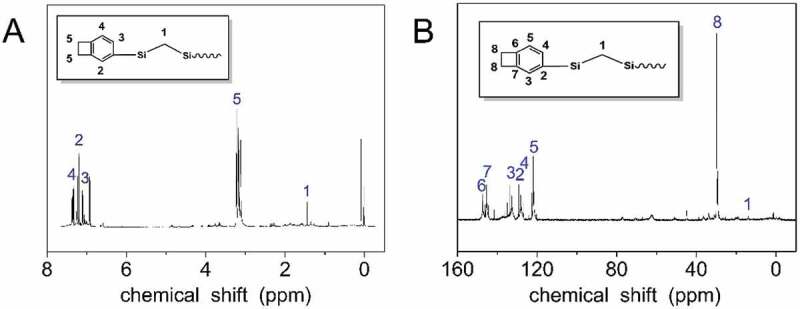
Structure characterization of HCBOs: A) ^1^H NMR spectrum; B) ^13^C NMR spectrum

The chemical structures of the HCBOs were confirmed by ^1^H and^13^C NMR. Figure 1A shows ^1^H NMR spectra of HCBOs. The occurrence of the hydrogen signal at approximately 7.11–7.33 and 3.22 ppm, which indicates the presence of phenyl and cyclobutene, demonstrates the incorporation of BCB. By integrating the peak area of the two type of hydrogens, the ratio of the hydrogen on the benzene of BCB to that on the four-member ring of BCB is 3:4, further indicating that the benzocyclobutene group has been successfully connected to the polycarbosilane. The hydrogen signal in the four-membered ring of benzocyclobutene appears in the range of 3.10–3.25 ppm, and the sum of the integral area is 3.69. The hydrogen signal of the benzene ring in benzocyclobutene appears in the range of 6.98–7.43 ppm, and the integral area is 1.05, 1.00, 0.75, respectively. The hydrogen signal of methylene unit in Si-CH_2_-Si appears in the range of 1.00–1.89 ppm with an integral area of 1.37. Thus, the ratio of methylene unit in Si-CH_2_-Si to benzocyclobutene unit is about 1:2.7. This ratio is consistent with the structural characteristics of hyperbranched products. Figure 1B shows the ^13^C NMR spectra of HCBOs. The occurrence of carbon signal at 121.94–147.34 ppm, which belongs to the six carbons corresponding to benzene ring in HCBOs. The carbon signal appeared on 29.88 ppm and was attributed to two carbon signal peaks in the four-membered ring of benzocyclobutene. Due to the symmetry of the two carbons, only one signal appears. Carbon signal appeared at 14.04 ppm, corresponding to the signal peak in HCBOs (Si-CH_2_-Si). The structure was further confirmed by ^13^C NMR spectra of HCBOs can be seen from Supporting Information.

**Figure 2.**
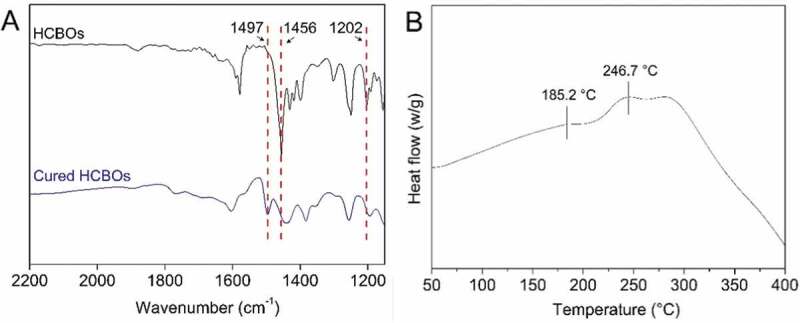
A) FTIR spectra of HCBOs before and after curing. B) DSC thermograms of HCBOs

Curing of HCBOs was characterized by FTIR spectroscopy. The characteristic absorption bands of BCB structures was found in FTIR spectrum of HCBOs (Figure 2A). The peaks at 1456 cm^−1^ and 1202 cm^−1^ were assigned to the characteristic absorption of in-plane bending vibration and out-plane bending vibration of the four-membered ring C-H bond on benzocyclobutene respectively, further confirming benzocyclobutene had been successfully grafted onto polycarbosilane structure. After thermal curing, the characteristic peaks at 1456 cm^−1^ and 1202 cm^−1^ were disappeared and a new peak appears at 1497 cm^−1^, which is the symmetric stretching vibration absorption peak of C-H bond on methylene after the ring-opening of the BCB quaternary ring. This indicates that the ring of the quaternary ring of benzocyclobutene has been ring-opened during the curing process, resulting in the formation of o-dimethylquinone intermediates. Subsequently, Diels-Alder reaction occurs between the two and the formation of cyclooctanediene in the middle of the benzene ring [23–25].

**Figure 3.**
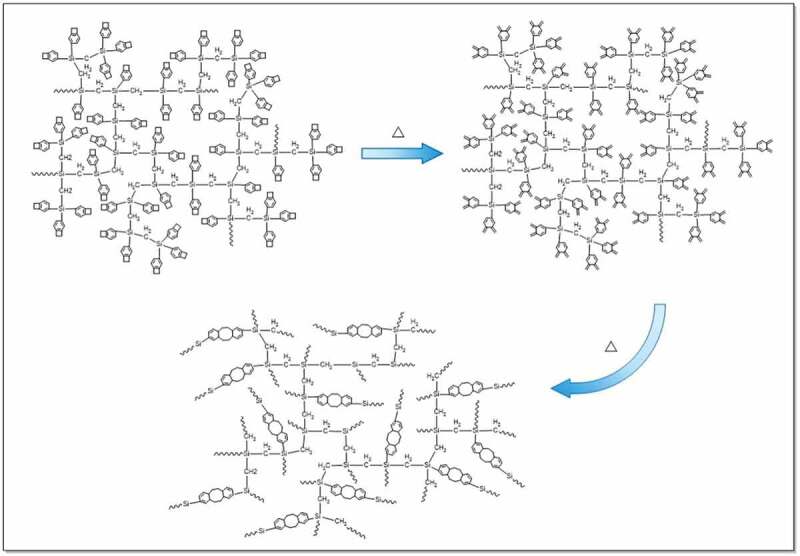
Curing reaction of HCBOs

Curing of HCBOs was further investigated by DSC as well. Figure 2B shows the differential scanning calorimetry (DSC) plots for the HCBOs recorded at a heating rate of 10°C/min. The obvious exothermic peak of the curve appeared at 185.2°C, and reached the maximum temperature at 246.7°C. The exothermic peak centered at around 246.7°C should be attributed to the ring-opening reaction of BCB. Such an exothermic feature was in good accordance with those of reported BCB-containing polymers or monomers [26–28]. In order to well understanding the curing mechanism of HCBOs at high temperature, a possible schematic procedure was depicted in Figure 3.

**Figure 4.**
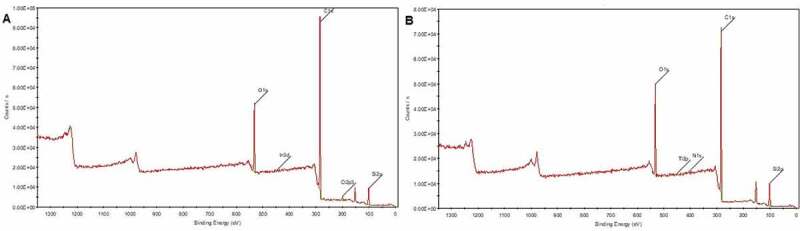
XPS survey: A) HCBOs after curing; B) BCBPCS after curing

**Figure 5.**
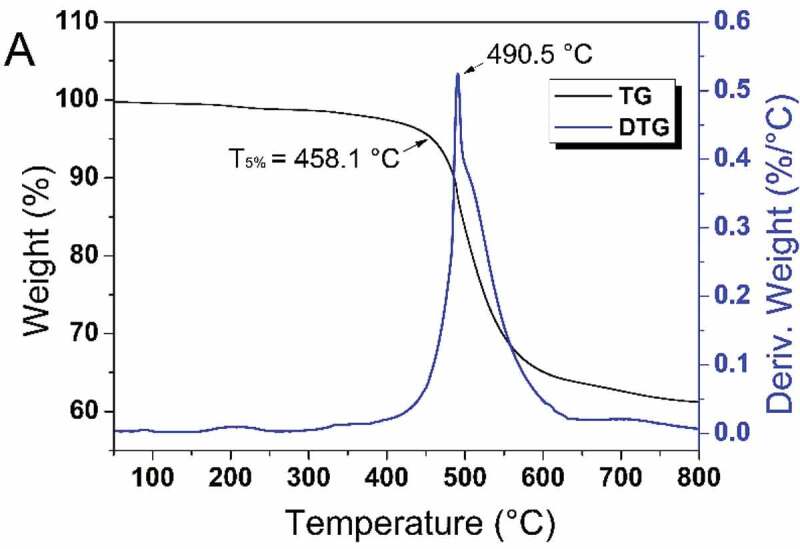
TGA and DTG curve of the cured HCBOs

Figure 4 shows the XPS survey of HCBOs and BCBPCS [19] after curing. It can be seen (Figure 4 and Table 1) that the C content of cured HCBOs is the highest as compared with Si and O elements. According to the binding energy of C, C exists in the form of organic matter. Moreover, it was found that C/Si ratio of HCBOs was appreciably higher than that of BCBPCS, indicating the existence of more Si-C bonds in HCBOs. Although the structure of HCBOs is mainly Si-C bond, but it is easy to hydrolyze and may be oxidized during thermal curing, resulting in the final cured product may containing Si-O groups, which is also the reason why there are O atoms in the composition analysis of XPS survey Figure 5.

**Table 1.** Ultimate analysis of HCBOs and BCBPCS

Sample	HCBOs	BCBPCS
C %	77.74	72.00
Si %	8.31	11.36
O %	12.37	15.19
C/Si	9.4	6.3

### 3.2 Thermal, dielectric, mechanical and surface morphology properties

The thermal properties of the cured resins were investigated by TGA. From the TGA curves, the T_5%_ of the cured HCBOs was around 458.1 °C and char yield was 61.2 % at 800 °C in a N_2_ atmosphere. The cured T_5%_ value is higher than the thermal decomposition temperature of most reported organosilicone resins (around 365 °C [29]). According to the DTG curve, the peak temperature at the point of maximum weight loss rate is 490.5 °C. The good thermal stability of HCBOs resins was attributed to the Si-C skeleton with highly branched (Si-C has a high bond energy) and cross-linked structure of the BCB group. The four-membered rings of BCB undergo a ring-opening reaction at high temperature to form stable cross-linked structure. As a result, HCBOs resins are able to withstand high-temperature processing conditions and meet the requirements of microelectronics packaging and other fields [30].

**Figure 6.**
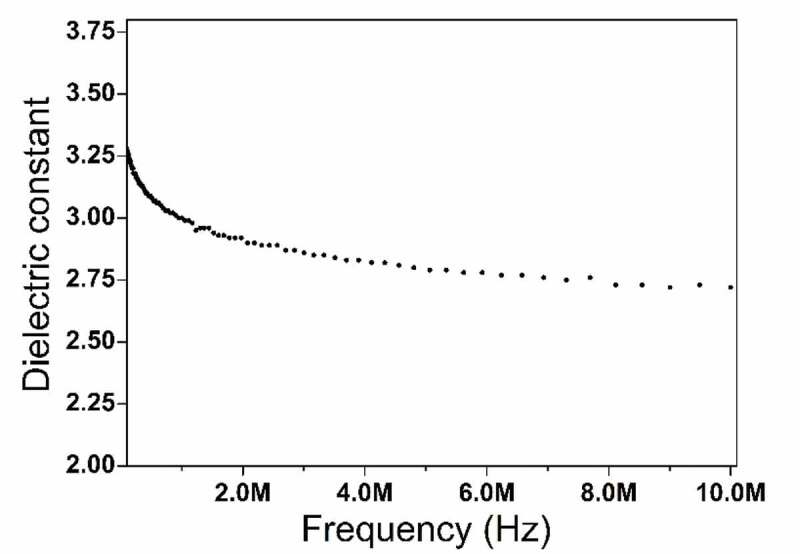
Dielectric constant curves with frequency of HCBOs

The dielectric constant of HCBOs resins was measured by impedance measurements at room temperature, and the results were illustrated in Fig.6. HCBOs showed a dielectric constant of 2.72 at the 10 MHz. It has good low dielectric properties and its value is lower than that of most commonly used and previously reported low-dielectric polymers (The dielectric constant is between 2.9 and 3.4 [31]). The reason why cured HCBOs shows good dielectric properties can be explained by the Debye equation [32], as shown in following:

$$\frac{k-1}{k+2}\;=\;\frac{4\pi }{3}\;N(a_{e}\;+\;a_{d}\;+\;\frac{u^{2}}{3K_{b}T})$$

Where *k* is dielectric constant, *N* is the number density of dipoles, *a_e_* is the electric polarization, *a_d_* is the distortion polarization, *u* is the orientation polarization related to the dipole moment, *K_b_* is the Boltzmann constant, and *T* is the temperature.

For HCBOs resins, the existence of Si-C bonds with low polarity made the polymers difficult to be polarized, resulting in low *a_e_*. The cross-linked structure prevented molecular stacking and distortion polarization, thus diminishing *N* and *a_d_*. As for cured HCBOs, it is an amorphous polymer. The *u* value of the amorphous structure is generally small given the low anisotropy. Thus, the reduction of all of the factors result in low dielectric constants for polymers. In addition, because of the ring opening of the four-membered ring of benzocyclobutene during thermal curing, the polymer is highly cross-linked and contains a large amount of intramolecular and intermolecular free volume, which increases the content of free volume in the curing system and is also conducive to the reduction of the dielectric constant. From comparing thermal and dielectric properties with other benzocyclobutene resins (Table 2), the cured HCBOs has good comprehensive performance, which suggest that the polymers can be used as encapsulation resins for integrated circuit (IC) molds or as laminated matrix resins for printed circuit boards.

**Table 2.** Thermal and dielectric properties of the polymers

	T_d5_	
Materials	(°C)	Dielectric constant
HCBOs	458	2.72
c-FSi-BCB [33]	453	2.60
BCB-Si-E [34]	400	2.77
P1 [35] P2 [36]	422 432	2.61 2.9

Nano indentation technology, also known as Depth-Sensing indentation (DSI), is one of the simplest methods to test the mechanical properties of materials. It is mainly used for testing the hardness and Young’s modulus of micro- and nanoscale thin film materials. The test results are calculated by the curve of force and pressing depth. The hardness and elastic modulus of the material are calculated as follows:

$$H=\frac{P_{max}}{A}$$

$$E_{r}=\frac{\sqrt{\pi }}{2\beta }\frac{S}{\sqrt{A}}$$

$$\frac{1}{E_{r}}=\frac{1-V^{2}}{E}+\frac{{1-V}_{i}^{2}}{E_{i}}$$

Where *P*_max_ is the maximum load applied, *E_r_* is reduced modulus (from elastic contact theory) [37–39], *E* and *V* are respectively the elastic modulus and poisson’s ratio of testing materials, *E_i_* and *V_i_* are the elastic modulus and poisson’s ratio of indenter respectively (the elastic modulus and poisson’s ratio of diamond indenter are *E_i_ *= 114 GPa and *V_i_ *= 0.07), *A* is the contact area.

**Figure 7.**
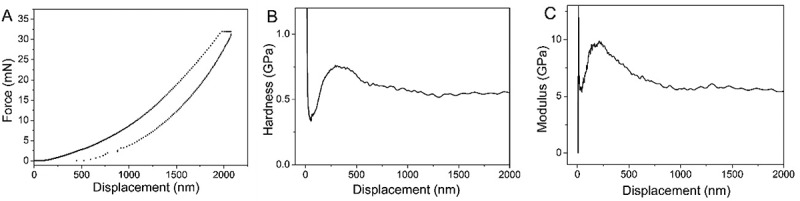
A).Curves of nano-indentation for HCBOs. B).The *d-H* curves of HCBOs. C).The *d-M* curves of HCBOs

Fig. 7 shows the relationship between the elastic modulus, hardness and the depth of indenter on HCBOs film. The hardness and elastic modulus of HCBOs always fluctuate in a certain range during the pressing process of the indenter. With the deepening of the pressing depth of the indenter (more than 250 nm), the fluctuation gradually tends to be gentle and the size tends to be constant. According to Fig. 7B-C, the average modulus of HCBOs is 7.1 GPa and the average hardness is 0.64 GPa.

The good mechanical properties of the cured products may be attributed to the rigidity of the chemical bond and the network connectivity of the polymer [40–42]. HCBOs contain the rigid group of benzene ring and the rigid Si-C bond. The ring-opening reaction occurs when four-membered ring of BCB is heated during the curing process, which further increases the crosslinking degree of the cured products and weakens the network connectivity in the polymer. The corresponding density of glass will be reduced accordingly. All these factors lead to high values of its modulus and hardness. The good mechanical properties indicate that it can be used in the fields of high performance or special materials which require high mechanical properties.

**Figure 8.**
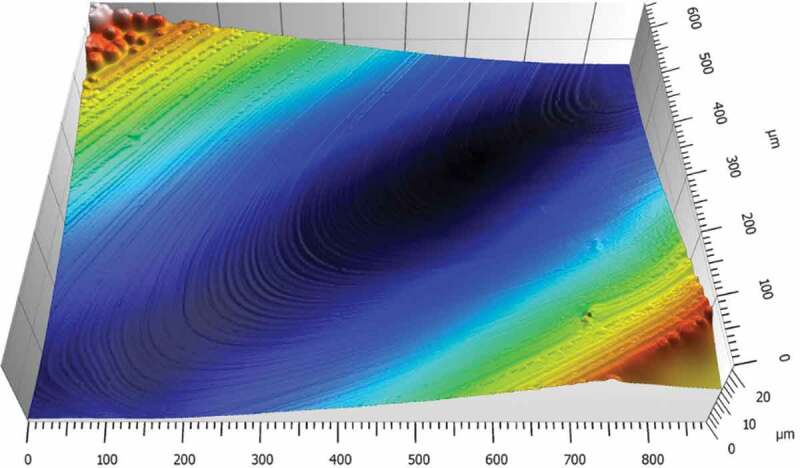
3D surface topography of the sample

The white light interferometer, also called non-contact 3D surface profilometer, is an ultra-precision surface profile measuring instrument based on the principle of optical interference. It is one of the most important methods to characterize the properties of thin films. It can provide the information of surface roughness, height and the 3D morphology of the material surface, and also obtain the discrepancy of the physical property distribution of the material surface [43]. It has been widely used in the surface morphology analysis of polymer materials. The surface morphology of the cross-linked polymer HCBOs film was studied and analyzed, as shown in Fig.8. The average surface roughness (Ra) and root-mean-square deviation of the surface (Sq) of HCBOs films are about 16.3 nm and 76.7 nm respectively, with good surface finish and high smoothness. The above results show that the cured HCBOs films have good properties of film formation and film retention. The good film properties suggest that HCBOs films have application potential as high-performance electronic materials.

## 4 Conclusions

In conclusion, new hyperbranched carbosilane oligomers (HCBOs) was prepared via Grignard coupling reaction using chloromethyltrichlorosilane as raw material. The resins of HCBOs can be converted into highly cross-linked polymers via the Diels–Alder reaction above 200 °C. As resulted cured resins possessed the combined excellent properties of easy processability, high thermal stability, good mechanics performance, good film-forming property and low dielectric constants. These properties enable its potential applications in the fields of semiconductor, integrated circuit, microelectronics and electrical industry.
